# The Human–Nature Experience: A Phenomenological-Psychoanalytic Perspective

**DOI:** 10.3389/fpsyg.2018.00969

**Published:** 2018-06-14

**Authors:** Robert D. Schweitzer, Harriet Glab, Eric Brymer

**Affiliations:** ^1^School of Psychology and Counselling, Queensland University of Technology, Brisbane, QLD, Australia; ^2^Institute for Sport, Physical Activity and Leisure, Leeds Beckett University, Leeds, United Kingdom

**Keywords:** phenomenology, natural world, nature, psychoanalysis, reflexivity, life-world

## Abstract

Drawing upon phenomenology and psychoanalytic concepts, we explore and explicate participants’ lived experience of the natural world. The authors draw upon Husserl’s description of consciousness as intentionality and his later work on the life-world, in exploring experiences which provide a basis for a psychochoanalytic understanding of the human–nature experience. Unstructured interviews were undertaken with nine participants, each of whom regarded nature as being significant for their sense of wellbeing. The lived experiences were explicated drawing upon the two processes: Giorgi’s descriptive phenomenological psychological methodology and psychoanalytic researcher reflexivity. Data analysis and explication involved the following steps: (1) a thorough reading of each interview transcript, (2) breaking data into parts by demarcating meaning units, (3) organizing data by translating meaning units into units of psychological experience through coding, and (4) arriving at a summary of the data which involved organizing and reviewing units of psychological experience. The process of reflection led to the formulation of an essential psychological structure of participants’ lived experience of the natural world. We argue that the human–nature relationship can be conceived in terms of psychoanalytic concepts, and in particular, constructs based upon an understanding of the primacy of attachment relationships. The natural world is elucidated as (a) nature being experienced as a primary attachment, (b) nature experienced as a secure base, (c) nature experienced as twinship, (d) nature experienced as containing, and (e) nature experienced as embodied. This paper extends previous empirical descriptions of the human–nature relationship by incorporating psychoanalytic processes and theory into a theoretically informed qualitative methodological stance. Beyond the traditional notion of nature as something ‘out there’ that we can interact with for cognitive or emotional restoration, participants in this study described the experience of nature as being integral to their sense of self. This study suggests that experiences that facilitate immersion in nature provide opportunities for the development of an integrated sense of self that has a profound impact on a participant’s sense of wellbeing. The findings further demonstrate the convergence between phenomenology and psychoanalytic constructs which offers a richness to our understanding the subjectivity of participants and their relationship with nature, a perspective not often attainable through more traditional quantitative research methodologies.

## Introduction

Our understanding of the relationship between human beings and the natural world has been of increasing interest to researchers over the past five decades. This is particularly evident in the proliferation of research exploring the effects of nature contact and feelings of connection to nature on human health and wellbeing, and environmental attitudes and behaviors. Greater proximity to, and feelings of connection with the natural world are seen to promote physical health, and psychological wellbeing including mood state, and community cohesion (Maas et al., 2009; Shanahan et al., 2016).

As we become increasingly engaged with a digital world, there is an argument that we have become more disconnected from the natural world. This disconnect from the physicality and enrichment associated with the natural world has impacts on both psychological and physical wellbeing where recreational sitting has been found to be related to raised mortality and cardiovascular disease risk (Stamatakis et al., 2011). Poignantly, the psychological and physical illnesses which characterize distress in many “advanced economies” are identified by some researchers as treatable in part by nature contact, for example, depression (Bratman et al., 2012; Shanahan et al., 2016), cardiovascular disease (Maas et al., 2009; Kardan et al., 2015), and symptoms of stress including high blood pressure (Brown et al., 2013). This paper aims to add to current perspectives on the human–nature relationship by exploring the lived experience of nature with individuals who regard their relationship with the natural world as important for their psychological health and wellbeing.Phenomenological interview data is further explicated through a lens of psychoanalytic theory.

### Wellbeing and the Nature-Human Relationship

For a number of decades, studies examining the nature-human relationship have found a positive relationship between experiences of nature and psychological health and wellbeing (e.g., Ulrich et al., 1991; Kaplan, 1995; Korpela et al., 2001, 2014). The major theoretical frameworks drawn upon to explain the observed link include Biophilia (Wilson, 1984), Attention restoration theory (ART) (Kaplan, 1993), Stress reduction theory (SRT) (Ulrich et al., 1991), and Place attachment theory (Giuliani and Feldman, 1993; Giuliani, 2003). Biophilia proposes that human beings have an innate affiliation with the natural world which is in turn fundamental to psychological and other domains of wellbeing (Kellert, 1997). From a SRT perspective interactions in natural environments reduce stress built up as a result of time spent in urban and everyday environments. Specifically, SRT claims that human beings have an evolutionary connection with nature and that specific characteristics of nature (complexity, depth, absence of threat) provide solice and the observed restorative benefits. While the SRT and Biophilic frameworks have made a considerable contribution to our understanding of the relationship between human beings and nature, critics point out that for a number of reasons these evolutionary notions do not stand up to scrutiny (Joye and van den Berg, 2011; Brymer et al., 2014). Attention restoration theory suggests that some environments are more conducive to restoring mental fatigue resulting from everyday urban lifestyles. Specifically, the attentional demands of everyday environments require deliberate focus which ultimately results in fatigue. The natural world on the other hand restores cognitive resources and the subsequent ability to focus because attention is held with reduced requirement of effort. A critical review of ART found only partial evidence for the efficacy of ART as an explanatory model (Ohly et al., 2016). While popular, ART might not be able to fully explain the genesis of wellbeing benefits arising out of the human–nature relationship (Hartig and Jahncke, 2017). Alternatively, an evolutionary perspective may be conceptualized in terms of cognitive processes, referred to a motivation and valuation (Mercado-Doménech et al., 2017). Motivation is thus a complex process involving both cognitive and implicit processes which play a part in the potential survival value of the human–nature process.

Place attachment theory is a multifaceted framework that proposes human beings develop emotional bonds with a real or imagined place. While not directly developed to explore the human–nature relationship from a wellbeing perspective the framework suggests that wellbeing can be enhanced through the effective interactions of individual characteristics and characteristics of particular places. Place attachment theory suggests that when compared to urban environments the natural world is rich in characteristics that facilitate positive emotional bonds and therefore wellbeing. Often these bonds are developed in childhood and brought forward into adulthood as central to individual experiences of wellbeing (Scannell and Gifford, 2010). However, strong bonds with place can also have negative impacts such as when competing needs for the same place result in conflict (Giuliani, 2003). Further, while place attachment theory has been linked to aspects of psychological wellbeing (Scannell and Gifford, 2016) the notion of ‘place’ in place attachment does not specifically refer to nature and might for example include home, even if home is heavily urbanized. The precise characteristics of nature that support positive emotional attachment with respect to psychological wellbeing are difficult to define.

There are a number of limitations to the above notions. For example, critics have pointed out that for the most part the above frameworks stem from positivist and cognitive notions and suggest that nature is a thing separate from human beings that impact on people and provide benefits to human beings (Seamon, 1982, 2000). From a phenomenological perspective the above notions adhere to Cartesian notions of ‘subject’ and object’ which fails to acknowledge the co-constitution of the experience of being-with or part-of nature (Schroeder, 2007). While the value of the theoretical frameworks should be recognized they might also be limited in their capacity to provide a complete explanation of the experience of wellbeing derived from engagement with the natural world (Seamon, 1982). For example, a phenomenological perspective appreciated the multi-sensory nature of reaching out in relation to the natural world (Schafer, 1977). The notion that soundscapes within urban environments have different qualities to soundscapes in natural environments has implications for wellbeing. Specifically, Schafer (1977) noted that human beings experience sounds in urban contexts as cluttered where individual sounds merge into a non-discernable noise. Sounds in natural environments on the other hand are individually clear and easily heard. From this perspective human auditory perception is attuned to information in natural soundscapes rather than urban soundscapes (Seamon, 1982). Phenomenology has long provided insights into the human–nature relationship that have implications for explicating the experience of wellbeing, most often through the investigations of human beings as embedded in place (e.g., Tuan, 1974; Relph, 1976). For example, Tuan’s notion of topophilia suggests that individual psychological wellbeing is linked to preferences for, and experiences of, specific types of place (Heimer, 2005). A study undertaken by Ogunseitan (2005) found strong links between those who scored high in a topophilia rating with psychological wellbeing with the presence of ecodiversity being most important. Despite the research undertaken from a phenomenological perspective that has explored the human–nature relationship and the implications for wellbeing from these findings little research has specifically set out to explore the phenomenology of the human–nature relationship from a psychological wellbeing perspective. The following section briefly discusses some of the conceptual and attitudinal overlap between phenomenology and psychoanalytic processes.

### Phenomenology and Psychoanalytic Theory

Dilthey (1991) argued that if we are to extend our understanding of being, human science must seek to examine phenomena from a place of humble inspection and invite *original fullness* and richness of experience. According to Dilthey, understanding or *verstehen* necessitates the employment of all our capacities, and in this way a *verstehen* science is distinguished from pure intellectual understanding or *verstand*. Phenomenology counters the deterministic heart of conventional empiricism, and seeks to “reflect on the visceral texture of experience, the sensuous perceiving of life, as it is ‘given’ to the experiencer, pregnant with layers of implicit meaning” (Finlay, 2011, p. 4).

The idea that phenomenology seeks to illuminate the layers of lived experience bears striking resemblance to psychoanalysis, which also seeks to explicate and understand the lived experience of the analysand through appropriation of his or her reality. For example, early in his writings, Freud (1915) identified the importance of neutrality, abstinence, and anonymity on behalf of the analyst. Embodiment of abstinence, anonymity, and neutrality in psychoanalysis protects against the imposition of the analyst’s own subjective view of reality and importantly, contains the analyst’s countertransference. In particular, neutrality on the part of the analyst ensures openness to new understandings of the analysand’s lived experience (Schafer, 1992). Similarly, Bion (1967) advocated beginning every analytic session “without memory or desire” to safeguard against the intrusion of the analyst’s own assumptions, preconceptions, and projections. In this regard, the analyst represents a convergence between the methodologies of phenomenology and psychoanalysis.

While not universally accepted, there is an argument that phenomenology and psychoanalytic theory are complementary, in that psychoanalytic theory and practice represents a science of human subjectivity. Wertz provides both an in-depth account of phenomenology as a science of consciousness addressing questions of meaning, values, and purpose and also the methodological overlap between phenomenology and psychoanalysis (Wertz, 1986, 2016). He suggests that to reject the analytic process and relegate psychoanalysis to the periphery of scientific methodology is to impede our understanding of human experience. In supporting a radical recognition of the limits of psychology as a quantitative discipline, mimicking the methods which have proven successful in the natural sciences, he laments that psychology “will remain lost in explanatory theory and affiliations with other sciences without methods capable of delivering him to … … the encounter with living persons” (Wertz, 1986, p. 599). The intersection of phenomenology and psychoanalysis thus provides a common pathway to explore human subjectivity. The current paper proposes an integrative process seeking a fuller understanding of human relatedness in the context of nature.

Psychoanalytic theory is an overarching term encompassing a range of perspectives, with contemporary theory being influenced by object relations and relational theorists, who adopt a two-person analytic perspective, and recognize the significance of intersubjectivity. That is, the caricature of the traditional analyst abiding by neutrality, has been replaced by an empathic approach sensitive to the “here-and-now” relationship between self and other. Furthermore, the approach is phenomenological, in the sense of privileging the immediate experience of the participants-in-relationship. This perspective has been incorporated into both self psychology, and the development of relational psychoanalysis. Self psychology prioritizes the integrity of the self, and draws upon constructs such as self integration, twinship, and mirroring, to explain the ways in which the individual achieves self integration. Relational psychoanalysis provides greater salience to the self-other relationship. The internalized templates deriving from these relationships, and the significance of these interpersonal experiences on psychic functioning are considered fundamental in constituting human experience (Mitchell and Aron, 1999). In contrast to the focus of classical psychoanalysis which privileged subjectivity and inner forces of the isolated mind, contemporary psychoanalysis privileges lived intersubjectivity. Key features of the approach, which guide our understanding of human phenomena include an appreciation of human development, with reference to the notions of embodiment, containment, and attachment, and ideas around the development of self, which is seen as potentially fragile, but achieves a sense of integration and coherence through our relationships. The relational perspective proposes that human experience can be understood in terms of projective identification, which in turn values counter transference as a key component of understanding “the other.” This notion is consistent with Husserl’s original emphasis upon the *Lebenswelt*, or lifeworld in which the direct experience of all players in human experience is valued.

Increasingly, psychoanalytic theory and processes are being incorporated into qualitative research (see Frosh et al., 2003; Midgley, 2006; Holmes, 2012). A number of studies have demonstrated the use of countertransference-inspired researcher reflexivity to illuminate aspects of human experience that may not emerge in traditional research interviews (see Clarke, 2002; Lucey et al., 2003), such as psychological defenses. For example, research by Walsh and Shulman (2007) suggested that splitting represents a useful construct to understand the ways in which migrants defend against the psychic pain associated with the loss of home, acculturative stress, and the task of restoring a sense of self.

Psychoanalytic interpretations of interview data, drawing upon contemporary perspectives, offers a novel perspective on the human relationship with the natural world and further, demonstrates the applicability of psychoanalytic theory to qualitative research. The aim of the current paper is to offer an enriched perspective on the lived human experience of the natural world, by drawing upon phenomenology and psychoanalytic constructs.

The current research draws upon countertransference-inspired researcher reflexivity to elucidate the nuances of the human–nature relationship. This involves drawing upon our own emotional response to interview data to appropriate participant’s lived experience of the natural world and make meaning of this experience. The position taken is perhaps best captured by child psychotherapist Alvarez (1985), in her writings on the notion of neutrality in the context of psychotherapy: “The achievement of sufficient distance from the patient to think, yet not so much distance that empathic sensitivity and counter-transference receptivity get lost” (Alvarez, 1985, p. 88). Countertransference, founded upon the concept of projective identification, provides a grounding for highlighting the experience of the researcher in accessing their own responses in arriving at an understanding of the other.

The integrative methodological approach used is inspired by the work of Wertz (1986, 2005), Finlay (2011), and Holmes (2012), all of whom advocate for thoughtful integration of research methodologies that are traditionally regarded as standalone, in the pursuit of understanding human experience. Whilst the application of psychoanalytic theory to psychological research is not entirely novel, this paper occupies unique ground in applying psychoanalytic theory to the lived human experience of the natural world.

## Materials and Methods

### Participants

Nine participants were interviewed as part of a larger study investigating the lived experience of nature (Glab, 2017, unpublished). Participants were recruited using a snowballing process, and all participants were over the age of 18 with the majority of participants aged between late 20 s and mid 30 s. Inclusion criteria required that participants needed to have lived experience of the natural world and to regard the natural world as being fundamental to their sense of health and wellbeing. Participation was voluntary.

### Data Collection Procedure

The study was approved by the QUT Human Ethics Committee. All participants gave written informed consent in accordance with the Declaration of Helsinki prior to interviews being conducted. Interviews were conducted in person (*n* = 7) or via video conference calls due to the physical location of participants at the time (*n* = 2). Interviews ranged from 40 to 110 min in length and were recorded on a digital audio recorder. Notes were made immediately following each interview, with regard to the researcher’s felt sense of interviewees, affective shifts, and other non-verbal cues observed during interviews. Interviews were then transcribed and explicated.

### Interview Process

Interviews were guided by openness, curiosity, and presence to what was being expressed by participants. Drawing upon Gallagher’s description of phenomenology as returning to “the thing themselves” and the primacy of experience of the lifeworld (Lebenswelt) (Gallagher, 2012), the interviewer sought to engage with the interviewee and structure the familiar as unfamiliar, and open for exploration. Specifically, interviewees were initially invited to describe their lived experience of the natural world (i.e., *please tell me about your lived experience of the natural world*). Open-ended, non-leading questions and prompts were used judiciously to clarify meaning or to encourage elaboration. Minimal encouragers, such as “please tell me more,” or “please elaborate” were used to convey the interviewer’s presence to participants. The interviewer prioritized the interviewee’s sense of safety in the interview and created, as far as was possible, a space within which they were encouraged to reflect on their experience of nature. A typical prompt may have been “we are interested in the immediacy of your experience, please tell us more.”

### Data Analysis

A two-stage process, drawing upon phenomenology and psychoanalytic theory, was used to explicate and make meaning of the transcribed data. The appropriateness of drawing upon both these traditions is well articulated by Wertz (1993) in which he argues for a convergence of these two traditions, pointing to the common commitment to the irreducible nature of mental life, the bracketing of theories and preconceptions, the necessity for self-reflection and empathy, and privileging a relational theory of meaning. The first stage, informed by Giorgi’s (2009, p. 2) descriptive phenomenological psychological method, which in turn draws upon Husserl’s development of phenomenology, involved a five stage process. These stages were: (1) collection of verbal data, (2) a thorough reading of each interview transcript, (3) breaking data into parts by demarcating meaning units, (4) organizing data by translating meaning units into units of psychological experience through coding, and (5) arriving at a summary of the data which involved organizing and reviewing units of psychological experience. This process of reflection led to the formulation of an essential psychological structure of the lived experience of the natural world.

The second stage of analysis involved an iterative process during which key themes identified through the initial phenomenological explication were reconsidered and reconceptualised from a psychodynamic stance. This process involved four stages: (a) reading through the data a second time, from a stance informed by relational psychodynamic theory and constructs, (b) noting both participant and researcher responses to the content of the data and the codes used to demarcate the data, (c) organizing overarching relational themes to reflect themes which emerge from the data drawing upon relational psychoanalytic theory (for example, *nature experienced as a primary attachment)*, and (d) explicating the data in relation to the overarching themes identified above. From this process, parallels between psychic and emotional experience of interpersonal relationships, and psychic experience and emotional aspects of the natural world were identified and explicated. Parallels that emerged as prominent are discussed below.

Methodological integrity was based upon the processes recommended by Levitt et al. (2017), involving: (a) fidelity to the subject matter, and (b) utility in achieving research goals. Fidelity to the subject matter required that the researchers were consistent during each of the two phases of the research, and maintaining allegiance to the phenomenon, in this instance, to the lived-experience of the participants. Similarly, understandings of psychoanalytic concepts were shared within the research team, to ensure fidelity to the constructs as developed within the theory which informed the second stage of the explication. Utility on achieving research goals involved the systematic application of the research methodology for each of the stages in the process of explication. This involved initially following the process articulated by Giorgi (2009), in his description of phenomenological research in psychology. The second stage involved the explicit adoption of the psychoanalytic stance in the explication of the findings. The validity of the reflexive process and subsequent theme development was ensured through a process of ongoing reflection and discussion between members of the research team during the data analytic process. The process continued until there was significant agreement on the emergent findings which formed the basis of the results of the study. During each of these stages, the researchers maintained a focus upon the specific research question which underpinned the study. Giorgi emphasizes the basic assumptions of phenomenology, with his focus on the notions of “bracketing”, intentionality, and rigor. He has thus been critical of alternative approaches which he regards as less rigorous, such as Interpretive Phenomenological Analysis (IPA), which he critiques on the basis of the philosophical foundations underpinning the approach and the lack of rigor associated with the analytic process (Giorgi, 2011). More specifically, Giorgi takes the view that the term “phenomenological” has been adopted by IPA theorists with little regard to the nature of “bracketing of the natural attitude” which is seen as fundamental to Husserl’s rendition of phenomenology nor the process of the phenomenological psychological reduction. He further criticizes IPA as being inductive as opposed to Husserl’s notion of phenomenology being intuitive and descriptive. In terms of the methodological process followed, Giorgi is critical of IPA’s “hesitation to proclaim fixed methods” which are seen as detracting from the scientific criteria of objectivity or intersubjectivity (Giorgi, 2011, p. 195).

## Results

The aim of this paper was to make meaning of the lived experience of wellbeing from experiences with the natural world through both a phenomenological descriptive stance and a psychoanalytic lens. Participants engaged in unstructured interviews guided by both phenomenological and psychoanalytic principles of curiosity, neutrality, and empathy. Researcher reflexivity played a key role making meaning of the lived experience as articulated by participants. This process led to a rich understanding which may not have emerged otherwise. Excerpts from interviews have been used to illustrate results. This process led to the identification of the following key themes (a) nature experienced as primary attachment; (b) natural world as secure base; (c) natural world as twinship; (d) natural world as containing environment; (e) natural world as sensory-emotional milieu.

### Researcher Observations of Interviewees

Making use of the interviewer’s own experience of participants during the interview process is privileged in both phenomenological and psychoanalytic approaches. Given that the current paper draws upon phenomenology and psychoanalytic theory, inclusion of the interviewer’s emotional experience of participants during interview is seen as adding value by more deeply illuminating participant feeling expressed within interviews. In his writings about the unconscious, Freud reflected on the manifestation of experience in psychoanalysis suggesting that significance of experience is often reflected in paradoxically small gesture or behaviors. He wrote: “Are there not very important things which can only reveal themselves, under certain conditions and certain times, by quite feeble indications?” (Freud, 1924, p. 27).

Participants were observed speaking about the natural world with profound feeling. Even before participants had given voice to lived experience of the natural world, their faces and bodies gave expression to their experience of the natural world. Participants were observed to close their eyes and smile as they reflected on their experience of the natural world. Some participants held their hands to their chests, while others were observed as subtly hugging themselves when remembering particular experiences with the natural world. Interviews were characterized by a reliable warmth that emanated from participants as they spoke about their respective experience of the natural world. Several participants were observed to hum as memories of the natural world were brought to consciousness. Two participants became tearful, and gesturing to their tears, articulated feeling overwhelmed by their affection for the natural world. When participants described experiencing a sense of vitality in the natural world, a parallel process was observed such that both participant and interviewer became animated and expressive, both in voice and physical movement.

Across interviews, participants described experiences of feeling held in the context of the natural world. The notion of a holding environment, originally conceived by Winnicott (1960), refers to the maternal provision of an environment meeting the needs of the entirely dependent infant. Holding refers not only to the physical holding or cradling of the infant, but also to sufficient meeting of needs that fosters continuity of being-in-the-world that abets integration and the development of a coherent self. Winnicott (1960, p. 47) wrote that the primary function of the holding environment is “the reduction to a minimum of impingements to which the infant must react with resultant annihilation of personal being.” Holding thus bears similarities to Heidegger’s (1962) notion of *das man* or they-self, such that the ‘I’ is experienced as indivisible from the world. Similarly, holding connotes an experience of oneness between mother and baby, in which the infant experiences himself as an extension of his mother, rather than separate to his mother.

The term *containment* is thus used interchangeably with *held* and *holding* as participants used both terms to describe their experience within nature. However, in contemporary psychoanalytic literature *containment* and *holding* refer to different developmental processes, though are commonly conflated (Wright, 2005). Bion (1962) in his description of containment conceives the infant as having awareness of his mother as being separate or outside of her or him -self. In the context of this paper, holding and containment are used interchangeably to describe the participant’s experience of self as integrated and coherent in the context of the natural world.

The following section describes the findings that emerged from the two-stage methodology previously described. Illustrative excerpts are taken from interview transcripts and provide the reader exemplars, which are then interrogated from a psychoanalytic perspective.

### Natural World Experienced as Primary Attachment

The experience of the natural world emerged as being experienced as a primary attachment. The term primary attachment, derived from object relations theory, refers to a primary “attachment figure” which in this case, is metaphorical (Wolf, 2002). This overarching theme emerged from the following natural meaning units: experience of nature as part of childhood; nature as nourishing; essential for individual wellbeing; longing for nature, and sense of loss at destruction of nature.

The notion of primary attachment is seen in the following excerpt:

*Well, you know they call it Mother Nature. That’s an appropriate term. It [natural world] is where I come from, so I’m connecting back to myself by connecting with nature because I came from it* (Jen, 30 years old).

The experience of the natural world emerged for participants as serving a similar function to an attachment figure or self object. In particular, the notion of a self object, which is core to human identity, captures the dynamic relationship as described by the participant. The natural world is experienced as a psychic artifact allowing the individual to recalibrate and gain a renewed sense of self. In the above excerpt, the participant articulates an experience of connecting back to herself when spending time with the natural world. Her description suggests that time spent away from the natural world may be experienced as time spent away from the self, and that a return to the natural world marks a return to the self. A number of participants suggested that the natural world may function as a good self object most often associated with childhood and adolescence, and remains part of their self structure into adulthood.

For some participants it is the significance of the natural world in meeting self object needs that makes the destruction of the natural environment particularly distressing, as illustrated in the following excerpt.

*When I was five, we moved to the outer suburbs of Brisbane, which then would have qualified as semi-rural. That area feels completely different now, and it feels painful to drive out there now. There are stands trees that are missing, houses where there were previously rolling hills speckled with horses, unmarked roads. I hate to see the space that has been left behind by trees removed, and replaced by bitumen or houses or fences. It reminds of me Avatar, the film, when they tear down the Home Tree, and the people are screaming. It’s like their hearts are on fire at the loss of this beautiful thing* (Hannah, 28 years old).

Hannah describes experiencing psychic pain in response to the urbanization and destruction of the area she grew up in. She finds it painful to expose herself to the visible signs of development and to the loss of the landscape of her childhood. Her experience of loss in response to destruction of the natural environment, which was described by several participants, reflects the traumatic loss of environmental self object support. For the individuals who participated in the current research, the natural world forms part of their endopsychic structure similarly to the way in which human self objects are part of emotional infrastructure. The natural world represents one of a suite of internalized objects, thus the loss of the natural world may evoke a similar kind of distress that one may experience at the loss of a human self object or attachment figure.

### Natural World Experienced as Secure Base

Attachment is founded upon the concept of a secure base, providing a grounding for human experience. The very notion of human experience being founded in the process of reflection draws upon the human capacity to make sense of lived experience, often occurring within the context of a sense of security in the relationship with the “other.” In the most primitive terms, this occurs at birth as the young baby attaches to the “other” for nurturance and safety, and continues throughout life. In the context of the current study, experience of the natural world as secure base is founded upon the following codes: experience of freedom in nature; return to nature; affording of play and exploration; nature as home, and sense of belonging in nature.

The overarching theme of the natural world being experienced as a secure base is expressed in the following:

*I think people talk about the natural world as something completely separate to us, but we are nature as well and I think we just forget that… Nature is like, ‘You’re welcome.’ It always feels like home. It really is a return to* (Daisy, 27 years old).

The above excerpt illuminates the natural world as indivisible from the embodied architecture of the individual. Similar to the previous excerpt, Daisy eschews the common perception of the natural world independent of self and references “collective forgetfulness.” In keeping with contemporary psychoanalytic conceptions of relationality, her language undermines notions of subject and object, as she speaks about the natural world as relationship, when she refers to the natural world as ‘home.’ The experience of both oneness and separateness with the natural world is reminiscent of early infantile experiences of attachment figures, in that the attachment figure functions as an extension of the infant self.

*It’s definitely that I need to relate to it [natural world] from when I wake up. If I haven’t got a window open because it’s too cold to get up or whatever, then it’s unnerving. I need to be able to see outside. I really relate to it from a feeling sense. I need to feel the sun beating down on me and hear the birds… It’s comforting to be able to hear it in the morning, to be able to hear the birds and see the leaves outside the window. I guess it’s like a homecoming, and it you’re removed from it for too long, then it becomes disconcerting* (Elle, 27 years old).

In this quote Elle expresses her need to relate to the natural world experientially as she becomes conscious of being awake. She describes her need to relate to the natural world as multi-sensory, and experiences separation from the natural world as unnerving. Her sensory attunement to the natural world as being experience-near alleviates anxiety. Needing to be in proximity to the natural world bears likeness to the concept of proximity seeking in attachment theory. Thus the participant seeks closeness to the natural world (i.e., the attachment figure), which provides a necessary and needed sense of comfort. Further, she has learned through experience that proximity to the natural world offers a sense of safety, best understood in terms of primal attachment needs. Repeated episodes of attachment figure availability, which in this case may be opening her bedroom window to hear the birds and look out into the tree canopy, leads to the development of self characterized by internal working models about self and others. Elle’s lived experience informs a direct perception of nature as reliable, comforting and secure.

### Natural World Experienced as Twinship

Twinship refers to a self psychology construct used to explain human development, and more commonly refers to a non-dualistic and primary desire of a young person, to feel alikeness with other human beings (Wolf, 2002). Over time, the individual is believed to tolerate greater differences between the self and “the other.” Similarly, interviewees reflected a parallel dynamic where participants expressed a desire to identify with nature experientially. This theme emerged from the following codes: experience of kinship with nature; love in relationship with nature; oneness with nature; self as part of nature, and nature as inspiring. These codes could be seen as cohering around a mutual finding process between person and nature. It is suggested that the experience of twinship between participant and the natural world can be understood as functioning similarly to twinship relatedness in an analytic dyad in meeting the participant’s need for essential alikeness, but without there being a mutual finding process.

*I am not separate from the intelligence of nature… the biology of my body holds the intelligence that I am revering in nature. The intelligence that knows how to maintain cells in my legs – I share that intelligence with nature* (Rebecca, 31 years old).

In the above excerpt, the participant gives voice to the shared, intelligence of the natural world and her own body. She regards the natural world and its inherent intelligence with reverence. She observes that the intelligence she admires in the natural world is of the same order as that of her own bodily intelligence. She finds herself *in* the intelligence of nature, because she *is* the intelligence of nature. The experience of finding oneself in another is one of the hallmarks of twinship. To find oneself in another, in the same way that a child may find herself in the face of her mother or in the gestures of his father, offers an experience of essential alikeness. Thus, the participant may be afforded an experience of admiration for her own biological intelligence – the same intelligence that she reveres in the natural world.

*I guess there’s a sense of being stripped back, brought back, returned to the world. I think that it’s kind of like, the intelligence that exists within nature – that is nature – is that which makes me possible. I experience a feeling of being kindred with nature* (Hannah, 28 years old).

Similar to the previous excerpt, the participant references an essential likeness between herself and the natural world. She articulates an experience of feeling kindred with the natural world, which in other words, may be expressed as feeling that she *is* nature *among* nature. Hannah’s experience of being nature among nature is not dissimilar to the self psychology concept of twinship, which Kohut (1984, p. 200) described as “confirmation of the feeling that one is a human being among other human beings.” However, in this case, rather than being human amongst humans, the twinship experience pertains to the feeling that one is nature among nature.

### Natural World Experienced as Containing

The notion of containment lies at the core of contemporary psychodynamic theory and practice, which refers in part, to the containment of the individual, that is, the process of providing a sense of safety as the person experiences emotional containment of their affective experiences, and also in the course of human development, where the parent, often the mother, provides a soothing environment for the child, and over time, the child is said to internalize this experience of containment (Wolf, 2002). In the current study, the notion of the natural world being experienced as containing was identified through the following codes: experience of nature as containing; nature as grounding; nature as perspective-giving; presence with nature, and vulnerability or sense of fear in nature. The overarching theme is evident in the following:

*I think the feeling range… the spectrum of feelings that you get in nature, and nature acts as a container for experiencing all those things. Almost like a therapeutic experience, it holds that space for you. And it’s only you to experience that, it’s not like you’re experiencing that with another human and having to navigate their feelings as well. It’s you with your feelings in that space* (Jen, 30 years old).

The above excerpt illuminates the natural world as a containing space in which the participant feels that she can experience a range of feelings without fear of a disproportionate, inappropriate or invalidating human response. She describes the natural world as offering a therapeutic experience, and gives voice to the idea that her space in the natural world is hers alone. There is no requirement for management of her own emotional experience, or the emotional experience of another human being, which is the aim of good psychotherapy. In the context of an analytic dyad, the patients’ experience is privileged with the analyst only offering his or her experience as a means by which to better understand the patients’ experience. It is the role of the analyst to provide a containing space for the patient to express his or her experience, without fear of criticism or consequence. Though in the context of the natural world there may be consequences for carelessness, the natural world represents an emotionally safe space akin to the therapeutic environment.

In her writings about the intersection of human experience and the natural world, Kiewa (1994) suggested that one of the benefits of spending time in the natural world is the concrete and immediate feedback from nature. She describes “the consequences of actions are even-handed in fundamentally different ways from those human interactions in other settings” (p. 187). In the current research, participants described experiencing a sense of safety when walking through forests or swimming in the ocean, despite possessing awareness of the dangers that exist in these natural environments. The following excerpt is illustrative:

*I guess there is this feeling of safety and reliability I guess. Like I know exactly how I would feel if I were among those woods… it’s as though nature is reliable in always being there… I mean, nature is inherently unpredictable in terms of weather and other natural phenomena, but it’s sort of predictably unpredictable. And there’s consistency in that* (Hannah, 28 years old).

Similarly, another participant described a feeling of safety in the natural world despite knowing that her safety is not guaranteed: “It’s safe. It’s an emotionally safe space, maybe it’s not physically safe all the time but it’s emotionally safe” (Lou, 28 years old).

These excerpts illuminate experiences of psychic safety (or containment) in the context of an otherwise unpredictable physical environment. Participants describe the natural world as a place of constant and reliable containment within which they experience themselves as held. Although the natural world may not offer the kind of conscious attainment that a mother may offer her child, there appears to be something about the reliability of the natural world that promotes a sense of containment.

### Natural World Experienced as Embodied

The natural world was experienced by participants as being primarily sensory and emotional, which we refer to as embodied. This mode of being-in-the-world was identified through the following codes: experience of cellular connection; urban claustrophobia; sensory experience in nature, and nature as felt. The overarching theme is well articulated in the following:

*I love when I just go from seeing trees and grass, to really seeing the grass and trees. Once I just decided to smell the ground and [laughs] it smelled amazing. I don’t know, I can only think that my relationship [with nature] is that I experience joy from interacting with nature, whether it be just laying on the grass and feeling the sun on my skin, and just like, soaking it in, in that moment* (Jen, 30 years old).

Another participant gave meaning to her experience of nature thus:

*It’s a complete sense of belonging. Like, ‘Ah, this is me. I remember now. I am from this, this is my home. It is like taking a beautiful, gentle breath and exhaling modern trappings. Sort of like cellular return. It kind of feels like my cells are returned to themselves, reminded of their beautiful simplicity within the context of the complexity of the whole* (Hannah, 28 years old).

The above excerpts are two of several, in which participants described sensory-emotional experiences with the natural world. Several participants gave voice to sensory experiences that were associated with feelings of familiarity, belonging, and of being known by the natural world. Kohut (1984) wrote:

*The mere presence of people in a child’s surroundings – their voices and body odors, the emotions they express, the noises they produce as they engage in human activities, the specific aroma of the food they prepare and eat – creates a security in the child, a sense of belonging and participating, that cannot be explained in terms of a mirroring response or a merger with ideals* (p. 200).

The rich sensory milieu of the natural world affords similar experiences of familiarity and comfort, particularly for individuals whose relationship with the natural world was forged during infancy and/or early childhood. Contemporary philosopher and author de Botton (2015) writes about the significance of sensory experience during childhood. He writes of one of the characters:

*The fundamentals of Esther’s childhood will be stored not so much in the events as in sensory memories: of being held close to someone’s chest, certain slants of light at particular times of day, of smells, types of biscuits, textures of carpet, the distant, incomprehensible, soothing sound of her parents’ voices in the car during long night-time drives, and an underlying feeling that she has a right to exist and reasons to go on to hope* (p. 110).

His description captures the visceral nature of early sensory experience, particularly in terms of those that evoke a sense of comfort and familiarity. It is suggested that similar experiences of comfort and belonging occur in the natural world, particularly for individuals whose relationship with the natural world has significant psychic import.

## Discussion

The aim of this paper was to explicate the lived human experience of the natural world using a novel two-stage analytic process. Data gathered as part of a larger phenomenological analysis was subjected to interrogation from a contemporary psychoanalytic perspective, with interview excerpts used to illustrate psychoanalytic interpretations of the human–nature relationship. The findings suggest that relationship with the natural world can be understood drawing upon common relational psychoanalytic concepts to make sense of participants’ lived experience of nature. The application of psychoanalytic theory to further interrogate phenomenological descriptions illuminated aspects of the natural world as being of significance in the development and maintenance of a healthy and coherent sense of self, particularly for individuals who identify as having a meaningful and ongoing relationship with the natural world.

The study draws upon phenomenological methodological principles with a view to explicating the lived-experience of nature. Both phenomenology and psychoanalysis are based upon an epistemology which seeks to gain an understanding of human experience. Drawing upon psychodynamic understandings provided an additional perspective, which we viewed as enriching our understanding of the experience of nature. That is, the natural world may be understood in terms of a primary attachment relationship, involving what object-relationship analysts call a good self object, or significant other, both in terms of felt experience and psychic importance. Participants consistently identified the natural world as a source of tranquility and comfort. The natural world was illuminated as a space in which a sense of belonging, cohesion, and containment was experienced. Collectively, participants described experiences of returning to self, homecoming, and familiarity with the natural world that restored psychic equilibrium. Drawing upon both a phenomenological and psychoanalytic perspective provides both insights into the life-world of the participants, not accessible through either framework on its own, and also demonstrates the feasibility of an emerging methodology characterized by the emergence of psychoanalytic and phenomenological theory, which in turn, share a common approach to the exploration of the life-world of the participant, and privileges and idiographic approach as an initial step in scholarly understanding of human experience (Wertz, 1986).

Participants identified that being with the natural world healed feelings of unease and rehabilitated an eroded sense of self, much like the embrace of a significant other. As Kohut (1984, p. 77) wrote of psychotherapy, “The essence of the psychoanalytic cure resides in a patient’s newly acquired ability to identify and seek out appropriate self objects as they present themselves in his realistic surroundings and to be sustained by them.” Participants articulated being able to recognize their need for immersion in the natural world after experiencing deterioration of self-continuity and self-cohesion.

Conscious engagement with the natural world may be understood by drawing upon psychotherapy constructs drawn from both contemporary object relations theory, and self psychology. In other words, the natural world offers a similarly validating experience, as discussed in the psychotherapy literature, in that the natural world neither interferes with, nor gratifies, nor casts aspersions about lived experience. For example:

[Regarding connecting with the natural world] *I guess it’s similar to when you really genuinely hug a person… and you take the time and we don’t do that with humans very often. I guess because we have so much other crap going on in our brains with other humans, but you don’t get that with nature. …. A tree is not going to talk to you or judge you* (Jen, 30 years old).

Whereas the fallibility of a human self object may lead to self object failure, the natural world simply is. To the extent that the natural world simply is, it cannot offer interpretations or actively participate in the promotion of psychic insight. We argue that the self is consolidated through a stable self-object bond with the natural world, particularly when the individual’s lived experience of the natural world is imbued with memory and positive associations facilitated by significant emotional involvement in the original event (Curci et al., 2015). Furthermore, there can be no interpretation or misinterpretation of the natural world as intending harm - it simply is. Arguably, a person may experience narcissistic injury in the form of failing to summit a peak, climb a tree, or navigate terrain. For example, if a person regards herself as physically capable or competent at navigating hostile terrain, and she is not able to demonstrate these skills to herself, she may experience psychic discomfort. However, in not having to account for the mind of the other as in interpersonal experiences, the task of making sense of this discomfort is simpler in the natural world.

The natural world may be experienced as restoring psychic equilibrium. It does not aggravate narcissistic injury nor does it threaten sense of self. It is experienced as predictably changeable, egalitarian, and uninterested in criticism or judgement. Nature is associated with nostalgia as the relationship is imbued with childhood memories, learning, and shared experiences with loved ones. It is a touchstone that we seek out to anchor ourselves and to restore our sense of self.

We propose that the notions presented in this study, drawing upon both phenomenology and contemporary psychoanalysis are particularly significant in the context of an increasingly distressed and often alienated population. In synthesizing the themes explicated in the study, we may see an analogy in Mahler’s notion of early symbiosis and the process of separation and individuation (Mahler et al., 1973). Of course, individuation is an important part of human development, where separation refers to the individual’s sense of identity. At the same time, through attachment, the infant internalizes the loving and approving “other” which in turn, contributes to successful social and emotional development and to healthy proximity seeking over the course of the person’s life. While the language of self psychology which has informed sections of the paper is sometimes clumsy, the paper has explored the ways in which relationship with nature may be experienced in terms of: primary attachment; as secure base; as twinship; as a containing environment; and as a sensory-emotional milieu. In each of these ways of relating, nature provides a basis for a “safe base” enabling the individual to explore and develop a sense of self in the confines of a safe relationship where ruptures may be attended to, managed and repaired as needed.

In essence, we have argued for the possibility that the natural world may function similarly to a secure attachment relationship, particularly in terms of the ways in which the individual experiences his or her self in the natural world, which in turn raises the importance of nature contact from an early age. Participants describe notions, such as feeling tranquil, relaxed and emotional restoration captured by Biophilia, ART, SRT, topophilia and place theories. However, the notions of topophilia and place as concepts are described in terms of nature out there and separate from humanity, places that we move to or through, places that facilitate emotional experiences. Equally, the notion of nature as something separate from humanity providing space to restore or realize emotional bonds has been effectively explored through Biophilia, ART and SRT. However, participants in this study indicate that, when focused on wellbeing, experiences of nature are beyond something out there and more than an emotional affiliation or a place to experience positive emotional or cognitive restoration. Instead, nature as expressed by those who experience wellbeing through nature, is experienced as family or part of self and in some way inseparable from self. Experiences of nature are described as contributing to an integrated sense of self. Participants sense of nature is multi-sensory and seems to reflect a comfortable attunement to information within the human–nature relationship which is often contrasted to human-human relationship. If an ongoing relationship with the natural world affords such a profound sense of belonging, comfort, and containment, there is even greater argument for immersive engagement with the natural world, particularly in the context of an increasingly nature-alienated global population.

### Limitations

Several limitations are noted. Application of psychoanalytic constructs to phenomenological data is novel. Traditionally, phenomenology rejects the application of theory to phenomena. Thus the task of harnessing both phenomenology and psychoanalytic theory toward explicating the lived experience of the natural world has required a two stage analytical process, during which lived experience has been identified, and the constructs, drawn from psychoanalytical constructs, have been utilized to make sense of the data.

The intersection of phenomenology and psychology is complex. Firstly, the convergence between phenomenology in psychological research and practice, and psychoanalytic concepts affords rich understanding of human experience. We are in agreement with scholars who have argued that this approach makes the nuances of experience accessible in ways not possible, either by methodologies based upon other disciplines, or a single approach such as phenomenology alone (Wertz, 1986). Secondly, this endeavor is inherently messy, intuitive rather than systematic, and thus replication can be difficult to achieve. However, our aim is to get close to human experience and to make sense of those experiences by drawing upon appropriate theoretical constructs. We have argued that contemporary psychoanalytical constructs are suitable for this purpose.

### Future Directions

The current findings suggest that the relationship between human beings and the natural world is significant, particularly in terms of psychic experience. The exploration of the human–nature relationship is particularly salient in the shadow of an increasingly disconnected global population. We argue for the need to continue to seek to understand the human experience of the natural world, and with this understanding, find ways to cultivate relationships between human beings and the rest of the natural world. It is not sufficient to know that nature contact is good for us - we already know this and yet the disconnect between contemporary sense of selfhood in urban environments and the natural world grows. Future research may benefit by focusing upon understanding the human–nature relationship, and use this insight to return to a fuller experience of our relationship with the natural world.

There is a need for integrative methodological approaches to further our understanding of human experience. While empirical methodologies may afford explanation of phenomena through postulation of abstract models and theories, phenomenology conceived as a human science lends itself to integrative models of enquiry. We have aimed to demonstrate that with alternative analytic procedures drawing upon phenomenological and psychoanalytic research, the vicissitudes of human experience may begin to be understood.

## Conclusion

This paper achieves two important objectives. First it demonstrates the utility of a novel methodology which draws upon both phenomenology as a rigorous descriptive science, and contemporary psychoanalytic theory and process to offer a rich and alternative perspective on a critically important relationship: our relationship with the natural world. Secondly, the findings extend our understanding of human experience as going beyond the traditional domains of early human-human attachment, and additional interpersonal relationships, which is at the center of much psychoanalytic reasoning, but as incorporating the relationship between human-beings and nature as being a profound component of human existence The use of researcher reflexivity to make meaning of the human–nature relationship illuminated parallels between relational psychoanalytic concepts and experience with the natural world. We argue further that the salience of the human–nature relationship, as articulated in this study may be of particular significance in the context of increasing mental health concerns and the rising incidence of chronic and stress-related disease. Encouraging deep and immediate relationships with the natural world may well represent one way of reinstating the centrality of nature in the lives of all human endeavor as we reclaim the term “mother nature.”

## Author Contributions

RS, HG, and EB were responsible for conceptualizing the study, were involved in the write up, and take responsibility for the final manuscript. RS provided training in the methodology and assisted with interviews. EB provided guidance to the field of environmental psychology. HG conducted the majority of the interviews, transcribed all interviews, and undertook the first analysis of all transcripts. EB and RS checked coding and analysis.

## Conflict of Interest Statement

The authors declare that the research was conducted in the absence of any commercial or financial relationships that could be construed as a potential conflict of interest. The reviewer SM and handling Editor declared their shared affiliation.

## References

[B1] Alvarez A. (1985). The problem of neutrality. *J. Child Psychother.* 11 87–103. 10.1080/00754178508254765

[B2] Bion W. R. (1962). *Learning from Experience.* London: Karnac.

[B3] Bion W. R. (1967). Notes on memory and desire. *Psychoanal. Forum* 2 272–273.

[B4] Bratman G. N. Hamilton P. J. Daily G. C. (2012). The impacts of nature experience on human cognitive function and mental health. *Ann. N. Y. Acad. Sci.* 1249 118–136. 10.1111/j.1749-6632.2011.06400.x 22320203

[B5] Brown D. K. Barton J. L. Gladwell V. F. (2013). Viewing nature scenes positively affects recovery of autonomic function following acute mental stress. *Environ. Sci. Technol.* 47 5562–5569. 10.1021/es305019p 23590163 PMC3699874

[B6] Brymer E. Davids K. Mallabon E. (2014). Understanding the psychological health and well-being benefits of physical activity in nature: an ecological dynamics analysis. *J. Ecopsychol.* 6 189–197. 10.1089/eco.2013.0110 26330207

[B7] Clarke S. (2002). Learning from experience, psycho-social research methods in the social sciences. *Qual. Res.* 2 173–195. 10.1177/146879410200200203

[B8] Curci A. Lanciano T. Maddalena C. Mastandrea S. Sartori G. (2015). Flashbulb memories of the Pope’s resignation: explicit and implicit measures across differing religious groups. *Memory* 23 529–544. 10.1080/09658211.2014.908923 24787135

[B9] de Botton A. (2015). *The Course of Love: A Novel.* London: Penguin Random House.

[B10] Dilthey W. (1991). *Introduction to the Human Sciences.* Princeton, NJ: Princeton University Press.

[B11] Finlay L. (2011). *Phenomenology for Therapists: Researching the Lived World.* Oxford: Wiley-Blackwell 10.1002/9781119975144

[B12] Freud S. (1915). *The Unconscious.* London: Hogarth Press.

[B13] Freud S. (1924). *A General Introduction to Psychoanalysis.* London: Hogarth Press.

[B14] Frosh S. Phoenix A. Pattman R. (2003). Taking a stand: using psychoanalysis to explore the positioning of subjects in discourse. *Br. J. Soc. Psychol.* 42 39–53. 10.1348/014466603763276117 12713755

[B15] Gallagher S. (2012). *Phenomenology.* London: Palgrave Macmillan 10.1057/9781137283801

[B16] Giorgi A. (2009). *The Descriptive Phenomenological Method in Psychology: A Modified Husserlian Approach.* Pittsburgh, PA: Duquesne University Press.

[B17] Giorgi A. (2011). IPA and science: a response to Jonathan smith. *J. Phenomenol. Psychol.* 42 195–216. 10.1163/156916211X599762

[B18] Giuliani M. V. (2003). “Theory of attachment and place attachment,” in *Psychological Theories for Environmental Issues*, eds Bonnes M. Lee T. Bonaiuto M. (Aldershot: Ashgate), 137–170.

[B19] Giuliani M. V. Feldman R. (1993). Place attachment in a developmental and cultural context. *J. Environ. Psychol.* 13 267–274. 10.1016/S0272-4944(05)80179-3

[B20] Hartig T. Jahncke H. (2017). Letter to the editor: attention restoration in natural environments: mixed mythical metaphors for meta-analysis. *J. Toxicol. Environ. Health B Crit. Rev.* 20 305–315. 10.1080/10937404.2017.1363101 28846499

[B21] Heidegger M. (1962). *Being and Time.* London: SCM Press.

[B22] Heimer H. (2005). Topophilia and quality of life: defining the ultimate restorative environment. *Environ. Health Perspect.* 113:A117 10.1289/ehp.113-a117

[B23] Holmes J. (2012). Using psychoanalysis in qualitative research: countertransference-informed researcher reflexivity and defence mechanisms in two interviews about migration. *Qual. Res. Psychol.* 10 160–173. 10.1080/14780887.2011.586451

[B24] Joye Y. van den Berg A. (2011). Is love for green in our genes? A critical analysis of evolutionary assumptions about restorative environments research. *Urban For. Urban Green.* 10 261–268. 10.1016/j.ufug.2011.07.004

[B25] Kaplan R. (1993). The role of nature in the context of the workplace. *Landsc. Urban Plan.* 26 193–201. 10.1016/0169-2046(93)90016-7

[B26] Kaplan S. (1995). The restorative benefits of nature: toward an integrative framework. *J. Environ. Psychol.* 15 169–182. 10.1016/0272-4944(95)90001-2

[B27] Kardan O. Gozdyra P. Misic B. Moola F. Palmer L. J. Paus T. (2015). Neighbourhood greenspace and health in a large urban centre. *Sci. Rep.* 5:11610. 10.1038/srep11610 26158911 PMC4497305

[B28] Kellert S. (1997). *Kinship to Mastery: Biophilia in Human Evolution and Development.* Washington, DC: Island Press.

[B29] Kiewa J. (1994). Self control: the key to adventure? Towards a model of adventure experience. *Women Ther.* 15 29–41. 10.1300/J015v15n03_04

[B30] Kohut H. (1984). *How Does Analysis Cure?.* Chicago, IL: University of Chicago Press 10.7208/chicago/9780226006147.001.0001

[B31] Korpela K. Borodulin K. Neuvonen M. Paronen O. Tyrväinen L. (2014). Analyzing the mediators between nature-based outdoor recreation and emotional well-being. *J. Environ. Psychol.* 37 1–7. 10.1016/j.jenvp.2013.11.003

[B32] Korpela K. M. Hartig T. Kaiser F. G. Fuhrer U. (2001). Restorative experience and self-regulation in favorite places. *Environ. Behav.* 33 572–589. 10.1016/j.amepre.2009.01.022 19269127

[B33] Levitt H. M. Motulsky S. L. Wertz F. J. Morrow S. L. Ponterotto J. G. (2017). Recommendations for designing and reviewing qualitative research in psychology: promoting methodological integrity. *Qual. Psychol.* 4 2–22. 10.1037/qup0000082

[B34] Lucey H. Melody J. Walkerdine V. (2003). Project 4: 21 transitions to womanhood: developing a psychosocial perspective in one longitudinal study. *Int. J. Soc. Res. Methodol.* 6 279–284. 10.1080/1364557032000091897

[B35] Maas J. Verheij R. A. de Vries S. Spreeuwenberg P. Schellevis F. G. Groenewegen P. P. (2009). Morbidity is related to a green living environment. *J. Epidemiol. Commun. Health* 63 967–973. 10.1136/jech.2008.079038 19833605

[B36] Mahler S. Pine M. M. Bergman F. A. (1973). *The Psychological Birth of the Human Infant.* New York, NY: Basic Books.

[B37] Mercado-Doménech S. J. Carrus G. Terán-Álvarez-Del-Rey A. Pirchio S. (2017). Valuation theory: an environmental, developmental and evolutionary psychological approach. Implications for the field of environmental education. *J. Educ. Cult. Psychol. Stud.* 16 77–97. 10.7358/ecps-2017-016-merc

[B38] Midgley N. (2006). Psychoanalysis and qualitative psychology: complementary or contradictory paradigms? *Qual. Res. Psychol.* 3 1–19. 10.1191/1478088706qp065oa

[B39] Mitchell S. A. Aron L. (1999). *Relational Psychoanalysis: The Emergence of a Tradition.* New York, NY: Analytic Press.

[B40] Ogunseitan O. A. (2005). Topophilia and the quality of life. *Environ. Health Perspect.* 113 143–148. 10.1289/ehp.7467 15687050 PMC1277856

[B41] Ohly H. White M. P. Wheeler B. W. Bethel A. Ukoumunne O. C. Nikolaou V. (2016). Attention restoration theory: a systematic review of the attention restoration potential of exposure to natural environments. Part B: critical reviews. *J. Toxicol. Environ. Health* 19 305–343. 10.1080/10937404.2016.1196155 27668460

[B42] Relph E. (1976). *Place and Placelessness.* London: Pion.

[B43] Scannell L. Gifford R. (2010). Defining place attachment: a tripartite organizing framework. *J. Environ. Psychol.* 30 1–10. 10.1016/j.jenvp.2009.09.006

[B44] Scannell L. Gifford R. (2016). Place attachment enhances psychological need satisfaction. *Environ. Behav.* 49 359–389. 10.1177/0013916516637648

[B45] Schafer R. (1992). *Retelling a Life: Narration and Dialogue in Psychoanalysis.* New York, NY: Basic Books.

[B46] Schafer R. M. (1977). *The Tuning of the World.* New York, NY: Knopf.

[B47] Schroeder H. W. (2007). Place experience, gestalt, and the human–nature relationship. *J. Environ. Psychol.* 27 293–309. 10.1016/j.jenvp.2007.07.001

[B48] Seamon D. (1982). The phenomenological contribution to environmental psychology. *J. Environ. Psychol.* 2 119–140. 10.1016/S0272-4944(82)80044-3

[B49] Seamon D. (2000). “A way of seeing people and place. theoretical perspectives,” in *Environment-Behavior Research: Underlying Assumptions, Research, and Methodologies*, eds Wapner S. Demick J. Yamamoto T. Minami H. (New York, NY: Kluwer Academic), 157–178. 10.1007/978-1-4615-4701-3_13

[B50] Shanahan D. F. Bush R. Gaston K. J. Lin B. B. Dean J. Barber E. (2016). Health benefits from nature experiences depend on dose. *Sci. Rep.* 6 1–10. 10.1038/srep28551 27334040 PMC4917833

[B51] Stamatakis E. Hamer M. Dunstan D. W. (2011). Screen-based entertainment time, all-cause mortality, and cardiovascular events: population-based study with ongoing mortality and hospital events follow-up. *J. Am. Coll. Cardiol.* 57 292–299. 10.1016/j.jacc.2010.05.065 21232666

[B52] Tuan Y.-F. (1974). *Topophilia: A Study of Environmental Perception, Attitudes, and Values.* Englewood Cliffs, NJ: Prentice-Hall.

[B53] Ulrich R. S. Simons R. F. Losito B. D. Fiorito E. Miles M. A. Zelson M. (1991). Stress recovery during exposure to natural and urban environments. *J. Environ. Psychol.* 11 201–230. 10.1016/S0272-4944(05)80184-7

[B54] Walsh S. Shulman S. (2007). Splits in the self following immigration. *Psychoanal. Psychol.* 24 355–372. 10.1037/0736-9735.24.2.355

[B55] Wertz F. (2005). Phenomenological research methods for counselling psychology. *J. Couns. Psychol.* 52 167–177. 10.1037/0022-0167.52.2.167

[B56] Wertz F. J. (1986). *Common Methodological Fundaments of the Analytic Procedures in Phenomenological and Psychoanalytic Research.* (New York, NY: Psychology Faculty Publications), 25.

[B57] Wertz F. J. (1993). The phenomenology of sigmund freud. *J. Phenomenol. Psychol.* 24 101–129. 10.1163/156916293X00099

[B58] Wertz F. J. (2016). Outline of the relationship among transcendental phenomenology, phenomenological psychology, and the sciences of persons. *Schutzian Res.* 8 139–162. 10.5840/schutz201688

[B59] Wilson E. O. (1984). *Biophilia: The Human Bond with Other Species.* Cambridge, MA: Harvard University Press.

[B60] Winnicott D. W. (1960). “Ego distortion in terms of true and false self,” in *The Maturational Process and the Facilitating Environment: Studies in the Theory of Emotional Development*, ed. Winnicott D. W. (New York, NY: International University Press), 140–152.

[B61] Wolf E. (2002). *Treating the Self: Elements of Clinical Self Psychology.* New York, NY: Guilford Press.

[B62] Wright K. (2005). The shaping of experience. *Br. J. Psychother.* 21 525–541. 10.1111/j.1752-0118.2005.tb00244.x

